# A new model for the treatment of type 2 diabetes mellitus based on rhythm regulations under the framework of psychosomatic medicine: a real-world study

**DOI:** 10.1038/s41598-023-28278-9

**Published:** 2023-01-19

**Authors:** Wenjiao Min, Xueli Sun, Nie Tang, Yaoyin Zhang, Fang Luo, Min Zhu, Wei Xia, Bo Zhou

**Affiliations:** 1 Psychosomatic department, Sichuan Provincial People’s Hospital, University of Electronic Science and Technology of China, Psychiatric Medical Center of Sichuan Province, Chinese Academy of Sciences Sichuan Translational Medicine Research Hospital, Chengdu, 610072 People’s Republic of China; 2 Mental Health Center, West China University Hospital, Sichuan University, Chengdu, 610041 People’s Republic of China; 3 Department of Endocrinology, Sichuan Provincial People’s Hospital, Chengdu, 610072 People’s Republic of China; 4 Psychosomatic department, The Fourth People’s Hospital of Chengdu, Chengdu, 610000 People’s Republic of China

**Keywords:** Drug discovery, Diseases, Endocrinology, Health care

## Abstract

We aimed to explore a new treatment model for type 2 diabetes mellitus (DM) based on rhythm regulation under the framework of psychosomatic medicine. Using psychotropics as rhythm regulators, 178 patients with DM were evaluated and divided into three groups: the antidiabetic treatment group (AT group), psychotropic treatment group (PT group), and combined antidiabetic + psychotropic treatment group (combined group), for a course of 16 weeks. The West China Psychiatry Association (WCPA) Somatic Symptom Classification Scale (SSCS) was used to evaluate each patient. The levels of hormones in the hypothalamic–pituitary–adrenal (HPA) and hypothalamic-pituitary-thyroid axes and of blood glucose and glycosylated hemoglobin (HbA1c) were evaluated both before and after treatment. After the treatment, the blood glucose and HbA1c levels in all three groups were lower than those at baseline. Furthermore, the incidence of the abnormal HPA axis in the PT group was significantly decreased (*P* = 0.003), while the incidence of the abnormal HPA axis in the combined group was 0.0%. The five factor scores of the SSCS in the PT and combined groups after treatment were both significantly low (*P* < 0.01). Both the incidence of abnormal neuroendocrine axes and SSCS scores in the AT group showed no significant difference before and after treatment. “Blood glucose control + rhythm regulation” should be considered as optimised treatment goals for DM. Moreover, some psychotropics could be used as biorhythm regulators, which have good potential value for clinical application.

Clinical trial registration number: ChiCTR1800019064. Name of trial registration: Reinterpretation of mechanism and the optimization of treatment for non-infectious chronic diseases under the “stress-dysrhythmia” theory hypothesis. The full trial protocol can be accessed at the Chinese Clinical Trial Registry (http://www.chictr.org.cn/).

## Introduction

Type 2 diabetes mellitus (DM) is a common chronic, non-infectious disease which exerts a heavy burden on the health care system^1^. The antidiabetic drugs used nowadays have certain shortcomings, one of which is a lack of sufficient preventive effects on the complications of DM^2^. DM is considered a typical psychosomatic disease as it often includes comorbid anxiety, depression, and insomnia^3^. Furthermore, changes in biological rhythms are closely related to DM, wherein patients often present with dysregulated neuroendocrine hormones, blood pressure, and blood glucose levels^4^. Previous studies have observed changes in the expression levels of some rhythm genes, such as *CLOCK* and *PER*, along with an increase in blood glucose levels. Moreover, mutations in these genes can lead to hyperglycemia^5^. In recent years, the relationship between biological rhythms, body health, and the occurrence of diseases has gained attention. Controlling blood glucose levels and improving systemic symptoms by regulating biorhythm could be a new treatment paradigm for DM^6^. A recent study found that metformin normalizes the abnormal circadian rhythm and upregulates the *CLOCK* gene in DM mice^7^. However, clinical studies are still lacking, and it is necessary to develop drugs which can potentially improve rhythmic regulation.

Bipolar disorder (BD) is considered a typical emotional rhythm disorder and is closely related to functional changes in the rhythm pathway^8^. Recently, our team has found that the distributions of single nucleotide polymorphisms (SNPs) of some rhythm genes, including ARNTL and PER, were significantly different in patients with type 2 DM or BD, suggesting that the genetic heterogeneity of biorhythm may be involved in the susceptibility to the two diseases^9^. Atypical antipsychotics, antidepressants, and mood stabilizers, including lithium and antiepileptics, are commonly used to treat BD. Many studies have found that these drugs act on the biorhythms of the body. For instance, lithium can directly affect the suprachiasmatic nucleus, increasing the self-excitation period of neurons^10^. Furthermore, quetiapine can change the mRNA expression levels of *BMAL1* and *PER1* genes in the amygdala^11^, while fluoxetine affects *CLOCK* expression in the hippocampus^12^. Therefore, we believe that these drugs have potential rhythm-regulating effects and could be ideal candidates for biorhythm-stabilizing therapy.

Taking the conventional treatment for type 2 DM as a control, this study used atypical antipsychotics, antidepressants, and mood stabilizers as potential rhythm regulators to explore a new treatment model of DM. This real-world study was aimed at optimizing the treatment of chronic non-infectious diseases, such as type 2 DM and providing clues for the development of new drugs.

## Methods

### Patients and treatment

DM patients, from southwest China, between January and December 2019 were enrolled in this study. The inclusion criteria were as follows: (1) Chinese Han origin; (2) aged ≥ 18 years old; (3) fulfilled the World Health Organization diagnostic criteria for type 2 DM (1999)^13^. The exclusion criteria were as follows: patients (1) with type 1 DM or other types of DM; (2) with acute or severe chronic complications; (3) who had used β-blockers or glucocorticoids in the previous 3 months; (4) with any history of other endocrine or autoimmune diseases; (5) with any history of major medical or neurological disorders including organic mental disorders, substance abuse, bipolar disorder, or schizophrenia; (6) who were pregnant or lactating; (7) who used more than two kinds of antidiabetic drugs; and (8) with history of insulin treatment.

All patients were of unrelated (no blood relationship) Chinese Han origin, sharing similar geographic and sociodemographic characteristics. This study was approved by the Institutional Ethics Committee of the Sichuan Provincial People’s Hospital. Written informed consent was obtained from all participants.

There were three groups in this study: the antidiabetic treatment group (AT group), psychotropic treatment group (PT group), and combined antidiabetics + psychotropics treatment group (combined group). After being fully informed, patients were enrolled in a certain group according to their own wishes, with reference to the doctor’s clinical evaluation. The course of treatment was 16 weeks.

Patients in the AT group used or continued to use conventional antidiabetics, while patients in the PT group were given only psychotropics. Patients in the combined group were treated with both conventional antidiabetic drugs and psychotropics. There were no limitations on the types and administration of drugs, and the treatment regimens were made by endocrinologists or psychiatrists after evaluations of the patients’ conditions.

A total of 190 patients with DM completed the 16-week treatment follow-up, 12 of whom were excluded owing to incomplete clinical data. Subsequently, a cohort of 178 patients was included in the analysis, with 88 in the AT group, 38 in the PT group, and 52 in the combined group.

Based on real-world clinical data, this study aimed to obtain a reliable and effective optimized treatment for DM.

### Data collection

Demographic and clinical data were collected after enrolment. The West China Psychiatry Association (WCPA) Somatic Symptom Classification Scale (SSCS) was used to assess somatic symptoms and their severities both before and after the treatment. The SSCS was developed by Zeng, Sun et al., Sichuan University. It consists of patients’ self-assessment and physician evaluation, with a total of 55 items, which are rated on a scale of 0 to 4, and includes five dimensions of inhibitory, irritable, biological, imaginative and cognitive somatic symptoms. The scale mainly evaluates the patients’ somatic symptoms and their influence in the last four weeks. The total score of items in each dimension divided by the number of items is the factor score of that dimension. The higher the score of a certain factor is, the more prominent the related symptoms are. The scale has a good reliability and validity with a α coefficient of 0.863 and a split reliability of 0.893^14^.

### Hormone measurements

Ten milliliters of peripheral venous blood was collected from each patient to measure the hormones of the hypothalamic–pituitary–adrenal (HPA) and hypothalamic–pituitary–thyroid (HPT) axes at 8:00 am, the day after enrolment, and the measurement was repeated at the end of the 16-week treatment period. The seven detected hormones were adrenocorticotrophic hormone (ACTH), cortisol (COR), thyrotropin-stimulating hormone (TSH), 3-triiodothyronine (TT3), thyroxine (TT4), free triiodothyronine (FT3), and free thyroxine (FT4). ACTH levels were measured using radioimmunoassay, and TSH levels were measured using the electrochemiluminescence double-antibody sandwich method. COR, TT3, TT4, FT3, and FT4 levels were measured by electrochemiluminescence quantitative assays. The normal ranges of the seven hormones levels is as follows: TSH: 0.27–4.2 mU/L, TT3: 1.3–3.1 nmol/L, FT3: 3.6–7.5 pmol/L, TT4: 62–164 nmol/L, FT4: 12–22 pmol/L, ACTH: 5.0–78 ng/L, COR (8:00 am): 147.3–609.3 nmol/L. Hormone levels above or below the reference range were both defined as “abnormal”. Patients were considered to have an abnormal HPA axis if they had at least one abnormal value of ACTH and COR levels, while they were considered to have an abnormal HPT axis if they had at least one abnormal value of TSH, TT3, FT3, TT4, and FT4 levels.

### Biochemical index measurements

Five milliliters of peripheral venous blood was collected from each patient the morning after enrolment to measure fasting blood-glucose (FBG) and glycosylated hemoglobin (HbA1c) levels. Each patient was then administered 75 g anhydrous glucose orally, and 2 h postprandial blood glucose levels (HPG) were measured. The FBG and HPG levels were monitored at the end of 4, 8, 12, and 16 weeks of treatment, while the HbA1c level was measured again after 16 weeks of treatment. Blood glucose levels were measured using a conventional biochemical analyzer (Johnson & Johnson), and HbA1c levels were measured using cation exchange chromatography.

### Statistical analysis

Sociodemographic data were analyzed using Pearson's χ^2^ test or one-way analysis of variance (ANOVA). Pearson’s χ^2^ test was used to compare the incidence of abnormal HPA or HPT axis among the three groups before and after treatment. The comparison of FBG, HPG, and HbA1c levels, and SSCS scores among groups was performed using ANOVA with Bonferroni correction for multiple comparisons. A non-parametric test was used if heterogeneity of variance was present. The comparison of abnormal HPA or HPT rates, FBG, HPG, and HbA1c levels, and SSCS scores before and after treatment in each group was performed either using Pearson's χ^2^ test or Student’s t-test. All tests were two-tailed, with alpha set at 0.05, and analysis was performed using SPSS 18.0.

## Results

### Comparisons of baseline data among the three groups

There was no significant difference in family history of DM and in hypertension among the three groups (both *P* > 0.05). The proportion of males in the AT group was significantly higher than the PT and combined groups (68.2%, 36.8%, and 38.5%, respectively, *P* < 0.001), and the age of the patients was also significantly higher in the AT group (60.57 ± 11.710 years) than the other two groups (50.42 ± 11.401 years and 59.62 ± 10.738 years, respectively, *P* < 0.001). Besides, the antidiabetic treatment course before enrollment among the three groups was also significantly different (χ^2^ = 6.412, *P* = 0.041), which was the longest in AT group (7.94 ± 7.399 years), followed by the combined group (5.48 ± 5.078) and shortest in PT group (4.34 ± 4.154).

**Figure 1:**
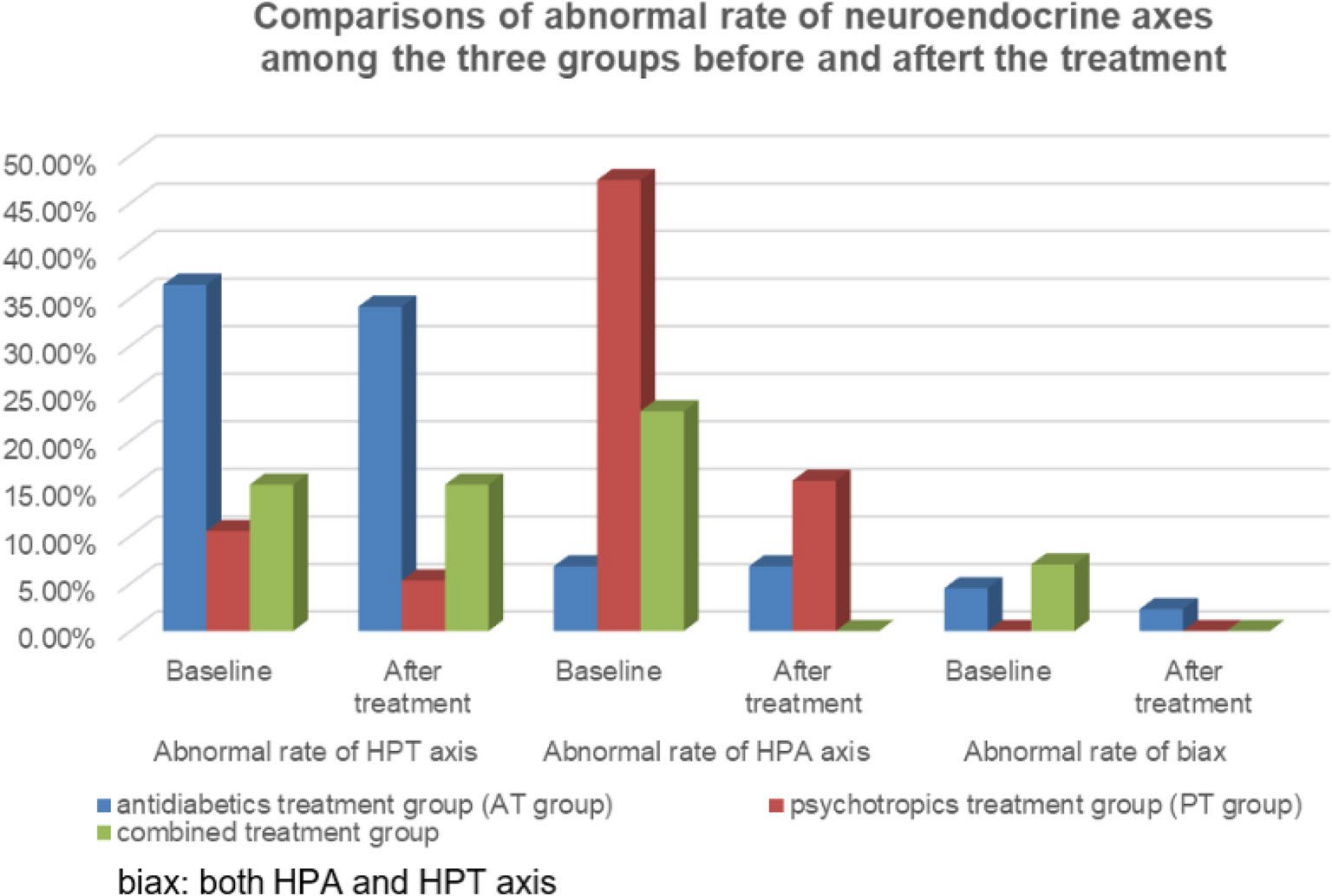
Comparisons of abnormal rate of neuroendocrine axes among the three groups before and after the treatment.

The baseline FBG, HBG, and HbA1c levels were significantly different among the three groups (*P* < 0.001) and all were highest in the AT group (Table 1). The incidence of abnormal HPT axis in the AT group (36.4%) was significantly higher than that in the other two groups (10.5% in the PT group and 15.4% in the combined group, χ^2^ = 12.960, *P* = 0.002), while the incidence of abnormal HPA axis in the PT group (47.4%) was significantly higher than that in the other two groups (6.8% in the AT group and 23.1% in the combined group, χ^2^ = 27.416, *P* = 0.000) (Fig. 1).

**Table 1:** Comparisons of blood glucose and HbA1c levels among the three groups before and after treatment.

Variables	FBG*		t_1_	P_1_	HPG^#^		t_2_	P_2_	HbA1c^@^		t_3_	P_3_
	A	B			A	B			A	B		
Groups												
AT group	10.19 ± 3.909	7.71 ± 1.714	5.444	0.000	16.34 ± 4.734	10.67 ± 2.548	9.884	0.000	9.44 ± 2.557	8.25 ± 1.788	3.580	0.000
PT group	7.39 ± 2.466	6.27 ± 1.318	2.477	0.016	10.91 ± 3.096	8.50 ± 2.201	3.916	0.000	6.83 ± 1.300	6.41 ± 0.796	1.702	0.093
Combined group	7.61 ± 3.758	6.01 ± 1.452	2.869	0.006	10.77 ± 3.334	8.17 ± 1.814	5.231	0.000	7.17 ± 0.962	6.43 ± 0.664	4.764	0.000
F	12.236	23.374			41.142	24.161			33.769	40.132		
P	0.000	0.000			0.000	0.000			0.000	0.000		

A: baseline; B: after the treatment.

P: Comparison among the three groups before and after treatment.

P1-P3: Self-control comparisons in each group.

*: Fasting blood-glucose.

^#^: 2 h postprandial blood glucose.

@: Glycosylated hemoglobin.

Except for biological somatic symptoms (*P* = 0.117), the baseline scores of inhibitory somatic symptoms, irritable somatic symptoms, imaginative somatic symptoms, and cognitive somatic symptoms of the SSCS were all significantly different among the three groups (*P* < 0.001). The scores of the above four types of symptoms were significantly lower in the AT group than those in the other two groups (corrected *P* < 0.001) (Table 2).

**Table 2:** The five factor-scores of SSCS among patients in different groups before and after treatment.

Groups	AT group		P_a_	PT group		P_p_	Combined group		P_c_	F/χ^2^_1_	P_1_	F/χ^2^_2_	P_2_
	A	B		A	B		A	B					
Variables													
Inhibitory	0.10 ± 0.031	0.08 ± 0.017	0.255	0.32 ± 0.229	0.08 ± 0.017	< 0.001	0.39 ± 0.253	0.09 ± 0.083	< 0.001	54.023	0.000	0.282	0.755
Irritable	0.33 ± 0.263	0.24 ± 0.219	0.023	0.66 ± 0.408	0.14 ± 0.134	< 0.001	0.78 ± 0.436	0.14 ± 0.125	< 0.001	43.102	0.000	13.539	0.001
Biological	0.48 ± 0.464	0.36 ± 0.351	0.059	0.27 ± 0.184	0.09 ± 0.027	0.003	0.39 ± 0.283	0.17 ± 0.133	< 0.001	4.290	0.117	26.292	0.000
Imaginative	0.09 ± 0.062	0.07 ± 0.039	0.425	0.47 ± 0.382	0.01 ± 0.071	0.004	0.49 ± 0.442	0.11 ± 0.015	< 0.001	54.641	0.000	1.947	0.146
Cognitive	0.16 ± 0.143	0.12 ± 0.114	0.021	0.42 ± 0.252	0.08 ± 0.054	< 0.001	0.36 ± 0.177	0.10 ± 0.153	< 0.001	52.252	0.000	12.825	0.002

A: baseline; B: after the treatment.

AT group: antidiabetics treatment group;

PT group: psychotropics treatment group.

Combined group: combined treatment group.

P_1_: Comparison among the three groups before the treatment.

P_2_: Comparison among the three groups after the treatment.

P_a_: Self-control comparison of PT group before and after the treatment.

P_p_: Self-control comparison of AT group before and after the treatment.

P_c_: Self-control comparison of combined group before and after the treatment.

### Comparisons of data after treatment among the three groups

FBG, HBG, and HbA1c levels after the treatment were significantly different among the three groups (*P* < 0.001), wherein all were highest in the AT group. The incidences of abnormal HPT (34.1% in the AT group, 5.3% in the PT group, and 15.4% in the combined group) and HPA axes (6.8% in the AT group, 15.8% in the PT group, and 0.0% in the combined group) after treatment were also significantly different among the three groups (χ^2^ = 12.960, *P* = 0.001 for the former; χ^2^ = 27.418, *P* = 0.013 for the latter) (Fig. 1).

The scores of irritable somatic symptoms, biological somatic symptoms, and cognitive somatic symptoms of the SSCS after the treatment were also significantly different among the three groups (*P* = 0.001, 0.000, and 0.002, respectively). The scores of the above three types of symptoms were significantly higher in the AT group than those in the other two groups (Table 2).

### Self-comparisons before and after the treatment

The levels of FBG, HBG, and HbA1c in the three groups after the 16-week treatment were significantly lower than the baseline levels (Table 1). The incidence of both the abnormal HPT and the HPA axes in the AT group was not significantly different before and after treatment (*P* > 0.05). On the contrary, the incidence of the abnormal HPA axis in the PT group significantly decreased after treatment (χ^2^ = 8.769, *P* = 0.003). Furthermore, the incidence of the abnormal HPT axis also showed a decreasing trend, but with no statistical significance (*P* = 0.093). The incidence of abnormal HPA axis in the combined group was 0.0%, which was significantly lower after treatment than that at baseline (χ^2^ = 14.452, *P* < 0.001) (Fig. 1).

The five factor scores of the SSCS in the PT group and combined group after the 16-week treatment were significantly lower than those at baseline (*P* < 0.01 for PT group, *P* < 0.001 for combined group), but there was no significant difference except irritable and cognitive factors in the AT group before and after treatment (*P* > 0.05) (Table 2).

### Comparisons between the PT and combined groups

The levels of FBG, HBG, and HbA1c, as well as factor scores of the SSCS, between PT and combined groups were similar at baseline and after treatment (*P* > 0.05). The incidence of abnormal HPA axis in the PT group was significantly higher than that in the combined group both at baseline (corrected *P* = 0.048) and after treatment (corrected *P* = 0.009).

### Comparisons of psychotropics use

Antidepressant monotherapy was the most commonly used treatment option in both the PT (47.4%) and the combined group (57.7%). The triple combination of atypical antipsychotics, antidepressants, and mood stabilizers was the second most used therapy in the PT group (26.30%), while the double combination of antipsychotics and antidepressants was the second most used therapy in the combined group (38.5%) (Table 3).

**Table 3:** The treatment options of psychotropics in different groups.

Treatment	Triple-combination	A + SGA	S + SGA	S + A	S	A	SGA
Groups							
PT group	26.30%	15.80%	5.30%	5.30%	0%	47.40%	0%
Combined group	0%	38.50%	0%	0%	0%	57.7%	3.80%
PT group	Antidepressants > triple-combination > antidepressants + SGA						
Combined group	Antidepressants > antidepressants + SGA > SGA						

A: antidepressants.

S: mood stabilizers.

SGA: atypical antipsychotics.

Triple-combination: antidepressants + mood stabilizers + atypical antipsychotics.

PT group: psychotropics treatment group.

Combined group: combined treatment group.

Table 4 lists the top three drugs used in the PT and combined groups for each psychotropic category. Escitalopram and sertraline were the most commonly used antidepressants in the PT (dose range: 5–10 mg/day) and combined (dose range: 50–200 mg/day) groups, respectively. Lamotrigine was the most commonly used mood stabilizer in the PT group (dose range: 25–75 mg/d). Lastly, olanzapine was the most commonly used atypical antipsychotic drug in both the PT and combined groups (dose range: 2.5–10 mg/d).

**Table 4:** The drug use and dosages in different groups.

	A	Usage rate (%)	Dosage (mg/d)	S	Usage rate (%)	Dosages (mg/d)	SGA	Usage rate (%)	Dosages (mg/d)
PT group	Escitalopram	36.8	5–10	Lamotrigine	21.1	25–75	Olanzapine	26.3	2.5–10
	Sertraline	21.1	50–150	Valproate	5.3	500–1500	Quetiapine	10.5	50–200
	Paroxetine	15.8	10–40				Aripiprazole	10.5	10–30
Combined group	Sertraline	38.5	50–200	–	–	–	Olanzapine	15.4	2.5–10
	Escitalopram	19.2	5–20	–	–	–	Quetiapine	15.4	50–100
	Venlafaxine	15.4	75–225	–	–	–	Paliperidone	7.7	3–6

Besides, Table 5 lists the top three antidiabetic drugs used in the AT and combined groups. Metformin was the most commonly used antidiabetic in both the two groups (dose range: 850–1700 mg/d in AT group, 500–1700 mg/d in combined group).

**Table 5:** The antidiabetic drug use and dosages in different groups.

	Group	Name	Usage rate (%)	Dosage (mg/d)	Group	Name	Usage rate (%)	Dosages (mg/d)
1	AT group	Metformin	22.73	850–1700	Combined group	Metformin	34.62	500–1700
2		Acarbose	18.18	75–150		Acarbose	15.38	75–150
3		Miglitol	11.36	50–100		Glimepiride	11.54	1–4

A: antidepressants.

S: mood stabilizers.

SGA: atypical antipsychotics.

PT group: psychotropics treatment group.

Combined group: combined treatment group.

AT group: antidiabetics treatment group.

## Discussion

To our knowledge, this is the first study to explore new treatments based on rhythm regulation in patients with DM. After 16 weeks of treatment, the levels of FBG, HBG, and HbA1c were significantly decreased in patients using either antidiabetics or drugs for rhythm regulation alone, suggesting that the rhythm regulation therapy was effective in blood glucose control. However, neuroendocrine axis abnormalities did not improve in patients treated with antidiabetics alone. In contrast, patients who received rhythm regulation therapy showed an improvement, suggesting that rhythm regulation therapy could not only control the blood glucose level but also improve the neuroendocrine axis functions. Dysfunction of the HPA axis and increased cortisol levels are closely associated with increased blood glucose levels and the risk for DM^15,16^. Therefore, this new treatment model might inhibit the development of DM and play a role in its prevention.

It is worth noting that the somatic symptoms in patients using antidiabetic medications were milder than in patients undergoing rhythm regulation therapy at baseline according to the SSCS assessment. However, after the treatment, the SSCS scores did not significantly improve in patients using antidiabetic medication, they even had significantly higher biological, irritable, and cognitive somatic symptoms than patients undergoing rhythm regulation therapy. Although the scores of inhibitory factor and cognitive factor in AT group decreased significantly after the treatment, the scores of the two factors in AT group were much lower before treatment than those in the other two groups, but much higher after the treatment. It suggested that the use of new treatment model can better improve patients’ somatic symptoms than traditional antidiabetic treatment. Patients with DM may have many non-specific somatic symptoms, which often impair their day-to-day function and is one of the main reasons to seek medical help^17^. The present study showed that treatment based on rhythm regulations could significantly relieve the somatic symptoms of patients with DM and improve quality of life, and it had a more optimized effect than the conventional therapy with antidiabetic medications.

In this study, patients in the combined group received both antidiabetic medications and rhythm regulation therapy, and their blood glucose and hormone levels as well as the severity of somatic symptoms at baseline were similar to those in patients who only received rhythm regulation therapy. After the 16-week treatment, the abnormalities of the HPA axis and the severity of somatic symptoms also showed similar improvements in the two groups. This suggests that the combined therapy did not have any added advantage over the single rhythm regulation therapy. Since the condition of patients enrolled in the two groups were relatively mild and those with severe complications were excluded, this result needs to be further confirmed with larger number of samples.

We found that compared with patients who received the new treatment model, those who only accepted antidiabetic therapy were older and predominantly males. In addition, the blood glucose and HbA1c levels, and the incidence of abnormal HPT axis in these patients were high. Previous studies have found that elderly individuals are conscientious^18^ and have relatively poor mental resilience under stress^19^. Furthermore, according to the three-stage theory of “General Adaptation Syndrome”^20^, a high HPT axis abnormality suggests severe physical damage to stress^21^. In addition, somatic symptoms were mild in these patients. Therefore, these three factors could explain why they were unwilling to accept the new treatment model. In the future, it will be necessary to include this group of patients to better explore the effects of rhythm regulation therapy.

Nowadays, the prevention of DM complications is also a very important issue in clinical practice. The deficiency of melatonin, which is closely related to circadian rhythm and sleep, was found to be associated with diabetic nephropathy (DN). It can activate the cardiovascular system and kidney receptors to protect from DN in preclinical models^22^. Besides, the animal research has found that type 2 diabetic(db/db) mice showed altered circadian patterns of both heart rate (HR) and sympathetic control of HR variability (HRV)^23^. As cardiac autonomic dysfunction is a serious complication of diabetes, we assume that treatment based on rhythm regulation may prevent the occurrence of this complication. Therefore, we will continue to observe the relationship between the use of psychotropics and diabetic complications, with monitoring the related rhythm indicators mentioned above, and further explore the exact relationship between psychotropics and rhythm regulation.

The antipsychotic drugs currently used mainly act on the dopamine system, and the *CLOCK* gene can affect the dopaminergic pathway^24^. Haloperidol and quetiapine at certain doses can increase the expression levels of *PER1, BMAL1*, and other rhythm genes^11,25^. Meanwhile, valproic acid can shorten the circadian behaviors in mice by reducing the expression of dopamine transporter. Valproic acid, which was thought to improve mood by normalizing the extended circadian cycle of patients experiencing elevated levels of dopamine due to decreased dopamine transporters, can also shorten the expression of the *CLOCK* gene in fibroblasts of patients with BD^26^. Studies have also shown that the circadian skin temperature rhythm of patients with depression became slow and returned to normal after antidepressant treatment^27^. Fluoxetine, a selective serotonin reuptake inhibitor (SSRI), can normalize *CLOCK* gene expression in mouse models of depression^12^. In addition, some variants of the *CLOCK* gene might be associated with the efficacy or side effects of SSRIs in depressed patients^28^. For example, patients carrying the C allele of rs1801260 in *CLOCK* showed a high incidence of insomnia during paroxetine or fluvoxamine therapy^29^. These previous studies support to some extent, the rationality of choosing atypical antipsychotics, antidepressants, and mood stabilizers as rhythm regulators.

Many researchers believe that it is necessary to pay attention to the psychological assessment and intervention of patients with DM^30^. Mind–body integrative therapy is particularly important, especially in patients with poor control of blood glucose and HbA1c, where the fluctuations of blood glucose might be a warning signal of stress in the body^31^. It is known that olanzapine is used with caution in clinical practice because it can increase blood glucose levels by modulating the genes related to glucose metabolism^32^. Interestingly, such an effect was not observed in this study, and olanzapine was the most frequently used atypical antipsychotic drug. This further confirmed that besides blood glucose control, the improvement of the stress-warning state in the body by rhythm regulation was also important in the treatment of DM. However, the association between increasing use of atypical antipsychotics us and the increased risk of incident DM has been concluded by previous researches^33^. Due to the short observation period of this study, it is not enough to fully explain the effects of drugs on metabolism. Therefore, it is necessary to conduct a long-term follow-up and comprehensive evaluation to further explore the value of antipsychotics in the treatment of DM.

Our previous study on the optimized treatment for atypical BD on a larger sample set showed that the triple combination of atypical antipsychotics, antidepressants, and mood stabilizers had a better effect than the double combination or single drug use, thus making it the preferred treatment model for rhythm regulation^34^. However, in this study, triple therapy was not the most commonly used therapy. Due to the small sample size and short treatment course, the comparison of the advantages of different treatment options was not powerful enough. We believe that the evaluation of therapeutic effects and monitoring of adverse drug reactions should be carried out in large samples and patients with severe complications should be included. This would allow us to further explore the optimized treatment of DM based on rhythm regulations in future studies. Moreover, the effectiveness of psychotropic drugs in the treatment of DM demonstrated in this study also supported, to some extent, that similar to BD, DM could also be considered as a rhythm disorder.

We recognize that there are some limitations with this study. First, due to the low acceptance by patients, the sample size, especially for the psychotropics treatment group was relatively small. Meanwhile, although we have excluded the DM patients with comorbidity bipolar disorder, depression, psychosis et al., adequate bias control was still lacking. As this was a real-world study, the results were more reliable and consistent with clinical practice. We will continue to expand the sample size and optimize the enrolled patients for observation in the future, as well as include DM patients with comorbid mental illness for comparison of treatment outcome. Second, a variety of drugs were included in this study, which may have confounding factors. Combination of antipsychotics, antidepressants, and mood stabilizers might mask the benefits of many drugs while combination of drugs itself could add confounding factors. However, the findings preliminarily reflect the differences of different treatment paradigm, which provide a basis for the next step of single drug effect comparison. In the next study, on the basis of expanding the sample size, we plan to compare the efficacy and safety of different drugs, so as to further clarify the therapeutic differences among them. Third, the expression rhythms of some rhythm genes may be affected by psychotropics^23^. Unfortunately, we didn’t detect gene expression in this study. Besides, although the normalization of hormones in HPA and HPT axis may indicate the improvement of neuroendocrine rhythm, the rhythmicity still needs further study. The diurnal rhythm changes of the neuroendocrine axis, such as cortisol diurnal rhythm, were not be monitored due to the condition limitations. We will supplement these indicators in the next study to provide more sufficient evidence for the rhythm regulation effect of psychotropics.

## Conclusion

In conclusion, this study preliminarily confirmed that psychotropics treatment for type 2 DM which may be based on rhythm regulations improves neuroendocrine functions, relieves somatic symptoms, and controls blood glucose levels. From this point of view, the optimized treatment goals for type 2 DM should be “blood glucose control + rhythm regulation”, where atypical antipsychotics, antidepressants, and mood stabilizers could be used as biorhythm regulators in future studies. However, these results still need be replicated in larger cohort to give a new direction in treatment of type 2 DM. This observation can potentially change the current situation, where patients with type 2 DM take antidiabetic medication for lifetime. Moreover, our results also provide some newe clues for drug research and development for clinical application.

## Data Availability

All data generated or analyzed during this study are included in this published article.
